# Treatment and survival outcomes of lobular carcinoma *in situ* of the breast: a SEER population based study

**DOI:** 10.18632/oncotarget.21461

**Published:** 2017-10-03

**Authors:** Pu Cheng, Qi Huang, Jiafeng Shou, Guoming Hu, Mengjiao Han, Jian Huang

**Affiliations:** ^1^ Department of Surgical Oncology, Second Affiliated Hospital of Zhejiang University School of Medicine, Hangzhou, China; ^2^ Department of General Surgery (Breast and Thyroid Surgery), Shaoxing People’s Hospital, Shaoxing Hospital of Zhejiang University, Zhejiang, China; ^3^ Department of Medical Oncology, Key Laboratory of Biotherapy in Zhejiang, Sir Runrun Shaw hospital, Medical School of Zhejiang University, Hangzhou, China; ^4^ Gastroenterology Institute, Zhejiang University School of Medicine, Hangzhou, China

**Keywords:** lobular carcinoma in situ, overall survival, lumpectomy, mastectomy, SEER

## Abstract

Lobular carcinoma *in situ* (LCIS) represents 5.3% of *in situ* specimens, and is thought to carry a low risk for developing to the invasive lobular breast cancer (ILC). There is still no standard care approach for patients with LCIS. We aimed to define the impacts of surgical and radiation intervention on survival outcomes of LCIS. LCIS cases from 2004 to 2013 of the recent Surveillance, Epidemiology, and End Results (SEER) database were analyzed. Clinicopathologic features were analyzed in 16002 patients between 2004 and 2013. Treatment modalities included no surgery (NS), lumpectomy alone (LA), lumpectomy with radiation treatment (LRT), mastectomy alone (MA) and mastectomy with radiation treatment (MRT). The overall survival (OS) was calculated by the Kaplan-Meier method. Univariate and multivariate analyses were performed using the variables of treatment, race, hormone receptor status, grade and age. Among 16002 patients, median follow-up was 54 months. Patients treated with LA had superior OS for NS (*P =* 0.001), MA (*P* < 0.001) and MRT *P =* 0.018). LRT only had superior OS for MRT (*P =* 0.009). There was no statistically significance between LA and LRT (*P =* 0.317). Improved OS was also correlated with younger age (*P* < 0.001), progesterone receptor positive (*P =* 0.001). Black patients had the worst OS (*P* < 0.001). There was no obvious survival difference among grade groups (*P =* 0.536). The LCIS patients treated with LA or LRT had better survival comparing with other groups. Considering the medical expense and the risk of radiotherapy, LA may be the most appropriate therapy for patients with LCIS.

## INTRODUCTION

Breast cancer *in situ* (BCIS) contains two distinct entities: lobular carcinoma *in situ* (LCIS) and ductal carcinoma *in situ* (DCIS). LCIS shares a number of similarities with atypical lobular hyperplasia. The primary difference between the two is the degree and extent of terminal duct and alveoli involvement [1–3]. LCIS is hard to be certain through present methods of medical examination, which can be definitively confirmed only by pathology [4, 5]. Mammography is the most sensitive imaging method in diagnosis of LCIS, with dotted microcalcification being the most common manifestation [6, 7].

Along with the constant popularization and improvement of breast cancer screening, incidence of BCIS has kept increasing [8]. It is reported that during the 1978–98 period, there had been a fourfold increase in the incidence of LCIS in America, from 0.90/100,000 to 3.19/100,000 [9]. And the proportion of patients diagnosed with LCIS in *open surgical biopsy* and core needle biopsy (CNB) lies between 0.5% and 3.8%, 0.02% and 3.8%, respectively [10, 11]. In developing countries, nonetheless, most patients visit hospital only after the clinical symptoms and signs show up [12]. Thus, it is difficult to get statistics on incidence of LCIS under present situation of such opportunistic screening in those countries.

Up to now, there is still much debate about the diagnosis and management of BCIS, particularly LCIS [13]. Women diagnosed with LCIS have a dramatically increased risk of invasive lobular cancer (ILC) and invasive ductal cancer (IDC) in either breast, with a relative risk that is eighteen and three to four times greater than that of the general population, respectively [6, 14]. Despite the risk increasing role of LCIS, some groups suggested that it could not be considered as a precursor of ILC [15–18] while others thought that it was quite another story [19–21]. In addition, some researchers stated that patients with LCIS were less susceptible to subsequent invasive breast cancer (IBC) in comparison with those with DCIS [22] while some others draw a contrary conclusion [21].

At present, there is no standardized treatment for LCIS. NCCN guidelines 2017 pointed out that surgical removal was suggested once LCIS was diagnosed via CNB [10]. However, some studies noted that the probability of pathological upgrading after surgical biopsy was only about one to five percent if results of CNB indicated non-high risk histopathological types [7, 23–25]. There were other authors reported that the local recurrence rate was not affected by positive resection margins during breast-conserving surgery [26]. Therefore, once diagnosis of LCIS is established via CNB, whether further excision is necessary or not is a matter for argument. As for radiotherapy, it is not recommended for application due to the absence of data support.

Great debate has hitherto existed on the treatment options of women diagnosed with LCIS. In the present study, impacts of various factors on survival outcomes for women with LCIS were analyzed using population-based data from the National Cancer Institute’s Surveillance, Epidemiology, and End Results (SEER) database. We especially gave prominence to the effect of different therapeutic methods on survival outcomes in the hope of finding an appropriate treatment for women with LCIS.

## RESULTS

### Incidence of lobular carcinoma *in situ* and cohort characteristics

**Figure 1:**
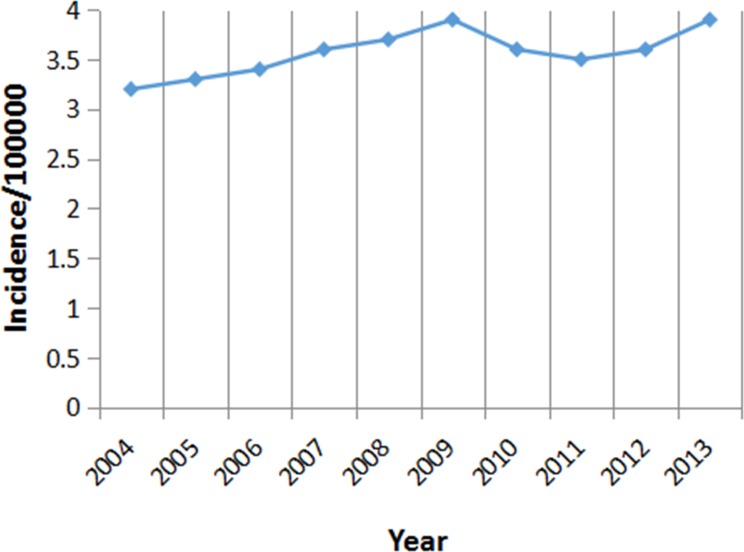
Incidence of LCIS between 2004 and 2013

As shown in Figure 1, the incidence of LCIS during 2004 to 2013 fluctuated between 3.2/100,000 and 3.9/100,000 in the United States, which showed an upward trend in the period between 2004 and 2009, following with a moderate decrease until 2011, and then started heading up again.

A total of 16002 women diagnosed with LCIS were included into our study. The most frequent treatment group was LA (68.1%), followed by the group of MA (21.4%), NS (9.1%), LRT (1.2%), and lastly MRT(0.2%). The baseline characteristics of each group were detailed in Table 1. No statistical difference in age was observed between five groups (*P* = 0.258). Statistical discrepancy existed between five groups in race (*P <* 0.001). White race represented the majority of cases in MA group (89.3%), while black race was demonstrated in only 5.8% (*n* = 200). Of all the patients, only 14.7% (*n* = 2356) had available information of hormone receptor status in the SEER database. Compared with other 3 groups, NS and LA groups had more remarkable differences in the distribution of hormone receptor status. Cases with positive ER/PR were 32 times (9.6% vs 0.3%) and 33 times (13.3% vs 0.4%) as many as those with negative ER/PR in NS and LA group, respectively. There were 1727 cases had integrated data of histopathological grade, accounting for only 10.8% of the total number of patients, with statistical differences between all the groups (*P <* 0.001).

**Table 1:** Baseline characteristics of included patients in the cohort (*N* = 16002)

Characteristic		NS	LA	LRT 198 (1.2%)	MA 3419 (21.4%)	MRT 27 (0.2%)	*P*
		1464 (9.1%)	10894 (68.1%)				
**Age [N (%)]**							0.258
< 40	473 (3.0%)	31 (2.1%)	326 (3.0%)	4 (2.0%)	111 (3.2%)	1 (3.7%)	
≥ 40	15529 (97.0%)	1433 (97.9%)	10568 (97.0%)	194 (98.0%)	3308 (96.8%)	26 (96.3%)	
**Race [N (%)]**							< 0.001
White	13593 (84.9%)	1155 (78.9%)	9192 (84.4%)	170 (85.9%)	3053 (89.3%)	23 (85.2%)	
Black	1267 (7.9%)	135 (9.2%)	912 (8.4%)	16 (8.1%)	200 (5.8%)	4 (14.8%)	
Asian/Pacific Islander	769 (4.8%)	58 (4.0%)	567 (5.2%)	12 (6.1%)	132 (3.9%)	0 (0%)	
American Indian/Alaska native	68 (0.4%)	10 (0.7%)	43 (0.4%)	0 (0%)	15 (0.4%)	0 (0%)	
Unknown	305 (1.9%)	106 (7.2%)	180 (1.7%)	0 (0%)	19 (0.6%)	0 (0%)	
**ER-PR status [N (%)]**							< 0.001
ER or PR Negative	2254 (14.1%)	140 (9.6%)	1450 (13.3%)	112 (56.6%)	548 (16.1%)	4 (14.8%)	
ER and PR Negative	102 (0.6%)	4 (0.3%)	48 (0.4%)	10 (5.1%)	39 (1.1%)	1 (3.7%)	
Unknown	13646 (85.3%)	1320 (90.1%)	9396 (86.3%)	76 (38.3%)	2832 (82.8%)	22 (81.5%)	
**Histopathological grade [N (%)]**							< 0.001
Grade I	730 (4.6%)	51 (3.5%)	488 (4.5%)	13 (6.6%)	178 (5.2%)	0 (0%)	
Grade II	758 (4.7%)	50 (3.4%)	514 (4.7%)	32 (16.2%)	158 (4.6%)	4 (14.8%)	
Grade III	206 (1.3%)	10 (0.7%)	101 (0.9%)	25 (12.6%)	70 (2.0%)	0 (0%)	
Grade IV	33 (0.2%)	1 (0%)	19 (0.2%)	5 (2.5%)	8 (0.2%)	0 (0%)	
Unknown	14275 (89.2%)	1352 (92.4%)	9772 (89.7%)	123 (62.1%)	3005 (87.9%)	23 (85.2%)	

Abbreviations: NS, No surgery; LA, Lumpectomy Alone; LRT, Lumpectomy-Radiotherapy; MA, Mastectomy Alone; MRT, Mastectomy-Radiotherapy.

### Survival outcomes

**Figure 2:**
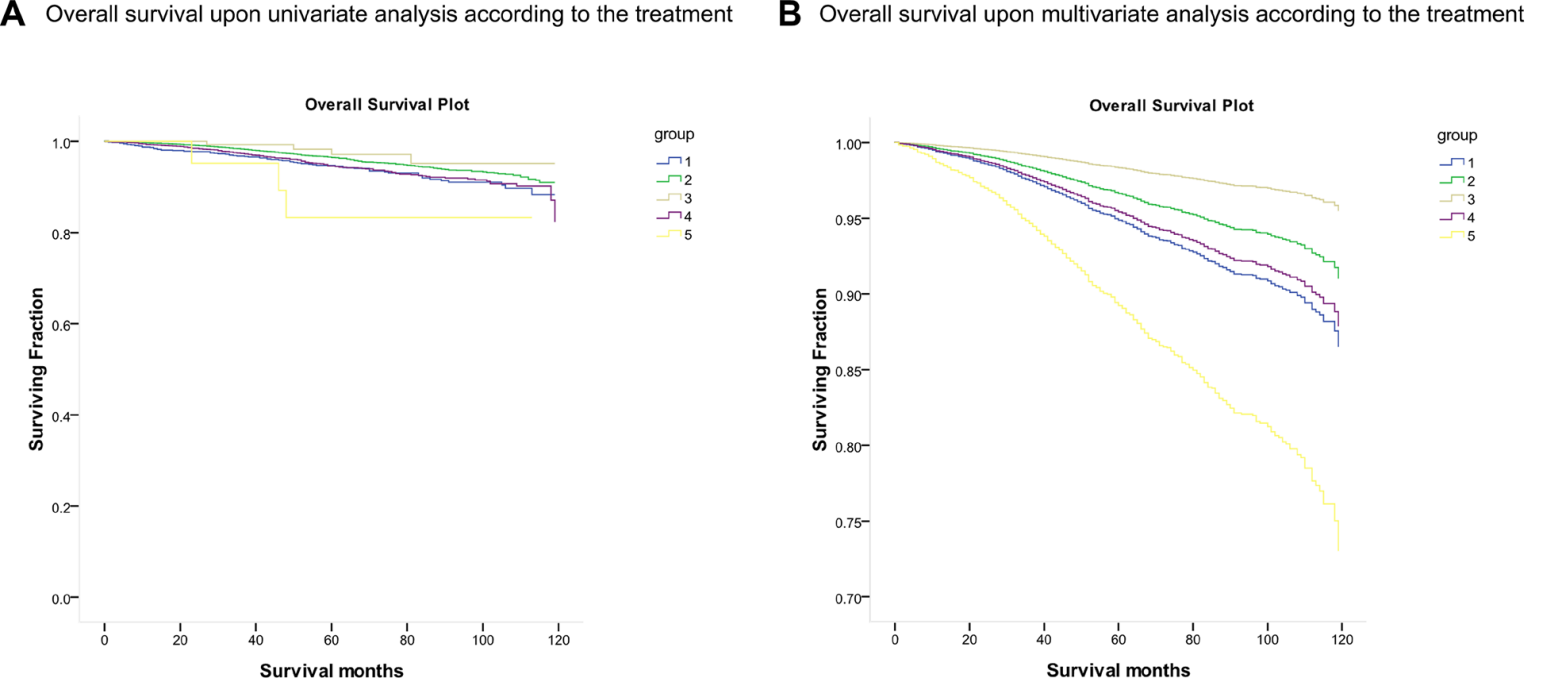
Kaplan–Meier curve of overall survival according to the treatment Kaplan–Meier curve of (**A**) Overall survival upon univariate analysis according to the treatment; (**B**) Overall survival upon multivariate analysis according to the treatment Group: (1) No surgery (NS); (2) Lumpectomy Alone (LA); (3) Lumpectomy-Radiotherapy (LRT); (4) Mastectomy Alone (MA); (5) Mastectomy- Radiotherapy (MRT).

Overall survival (OS) was compared according to different factors (Figure 2A). Results of univariate survival analysis (*log*-*rank* testing) were shown in Table 2. Compared with other treatments, LRT was able to achieve longer average OS (LRT, 116.242 months; NS, 112.479 months; LA, 114.601 months; MA, 113.041 moths; MRT, 100.857 months). *P* values for comparisons between LA group and other groups were significant (NS, *P* = 0.001; MA, *P <* 0.001; MRT, *P* = 0.018) except for the comparison with LRT group (*P* = 0.317). LRT only had survival advantage over MRT (*P* = 0.009). Patients diagnosed with LCIS at an earlier age (< 40) had longer average lifespan than those at ≥ 40 (*P <* 0.001). Relative to other races, black race and American Indian/Alaska native had poorer prognosis (*P <* 0.001). As for hormone receptor status, PR positive patients had longer average survival time than PR negative cases (*P <* 0.001). However, ER status had no pronounced influence on OS (*P* = 0.150). And we also found there was no remarkable effect of histopathological grade on OS (*P* = 0.536).

**Table 2:** Univariate survival analysis comparing OS in women with LCIS

Characteristic	Average survival time (Month)	95% CI	*P*
**Treatment**			< 0.001
NS	112.479	(111.048, 113.910)	Ref
LA	114.601	(114.173, 115.030)	0.001
LRT	116.242	(113.591, 118.894)	0.061
MA	113.041	(112.102, 113.980)	0.513
MRT	100.857	(88.238, 113.476)	0.151
**Age at diagnosis**			< 0.001
< 40	118.205	(117.309, 119.101)	Ref
≥ 40	113.979	(113.592, 114.367)	< 0.001
**Race**			< 0.001
White	114.182	(113.779, 114.584)	Ref
Black	111.665	(110.025, 113.306)	0.001
Asian/Pacific Islander	115.865	(114.347, 117.383)	0.341
American Indian/Alaska native	107.908	(101.306, 114.511)	0.06
Unknown	117.529	(116.608, 118.450)	0.013
**ER**			0.150
Positive	113.45	(112.330, 114.569)	Ref
Negative	110.275	(105.499, 115.051)	0.508
Unknown	114.238	(113.837, 114.638)	0.1
**PR**			0.001
Positive	113.828	(112.637, 115.020)	Ref
Negative	109.743	(106.379, 113.108)	0.008
Unknown	114.262	(113.863, 114.660)	0.36
**Histopathological grade**			0.536
Grade I	115.156	(113.509, 116.802)	Ref
Grade II	114.052	(112.266, 115.838)	0.352
Grade III	111.594	(107.877, 115.310)	0.097
Grade IV	101.754	(94.775, 108.733)	0.388
Unknown	114.092	(113.693, 114.491)	0.249

Abbreviations: Ref, reference; CI, confidence interval.

MVA for factors affecting prognosis on the basis of UVA results was then conducted. Given the clinical importance of ER status and histopathological grade, both of the two were also included for MVA. Kaplan–Meier curve of OS for MVA was displayed in Figure 2B. Treatment was an important predictor of OS, with patients underwent LRT (HR = 0.147, 95% CI 0.033–0.663, *P =* 0.013) and LA (HR = 0.300, 95% CI 0.096–0.935, *P =* 0.038) demonstrating better survival compared to those who accepted MRT. In contrast, neither NS (*P =* 0.190) nor MA (*P =* 0.129) had great effect on OS compared to MRT. Age at diagnosis was another factor associated with better OS, which was characterized by the better prognosis of patients diagnosed with LCIS at age < 40 (HR = 0.162, 95% CI 0.052–0.504, *P* = 0.002). In addition, it was revealed that poor prognostic significance was relevant to the black race (HR = 2.346, 95% CI 1.362–4.041, *P* = 0.002). Corresponding to the UVA results, PR positive patients showed better OS than PR negative ones (HR = 0.513, 95% CI 0.312–0.843 *P* = 0.008), while no significant effects on OS of ER status (*P* = 0.335) nor histopathological grade (*P* = 0.562) was observed. Results of *MVA* were detailed in Table 3.

**Table 3:** Multivariate survival analysis comparing OS in women with LCIS

Characteristic	HR	95% CI	*P*
**Treatment**			< 0.001
NS	0.462	(0.145, 1.466)	0.19
LA	0.3	(0.096, 0.935)	0.038
LRT	0.147	(0.033, 0.663)	0.013
MA	0.412	(0.131, 1.293)	0.129
MRT	Ref		
**Age at diagnosis**			0.002
< 40	0.162	(0.052, 0.504)	0.002
≥ 40	Ref		
**Race**			< 0.001
White	1.51	(0.917, 2.486)	0.105
Black	2.346	(1.362, 4.041)	0.002
Asian/Pacific Islander	2.323	(0.776, 6.954)	0.132
American Indian/Alaska native	Ref		
Unknown	0.181	(0.024, 1.369)	0.098
**ER**			0.335
Positive	1.421	(0.605, 3.341)	0.42
Negative	Ref		
Unknown	3.307	(0.676, 16.170)	0.14
**PR**			0.008
Positive	0.513	(0.312, 0.843)	0.008
Negative	Ref		
Unknown	0.195	(0.047, 0.803)	0.024
**Histopathological grade**			0.562
Grade I	0.453	(0.105, 1.952)	0.288
Grade II	0.597	(0.142, 2.523)	0.483
Grade III	0.8	(0.177, 3.619)	0.772
Grade IV	Ref		
Unknown	0.605	(0.150, 2.447)	0.481

Abbreviations: Ref, reference; HR, hazard ratio; CI, confidence interval.

## DISCUSSION

It has been well-established that, like DCIS, LCIS is a risk factor for subsequent IBC. However, there is a dispute over the hypothesis that LCIS is a precursor of ILC [15–21]. Up to now, no consensus has been reached clinically on the treatment of LCIS. And few large cohort studies have evaluated the clinicopathologic features of LCIS patients receiving different treatments.

In this SEER population based study, patients receiving LA reached the majority to 68.1% of the entire cohort, while MA, once the first choice, made up 21.4%. This indicated that, for patients with LCIS, most clinician inclined to perform surgery rather than select non-operative management. The rates of pathological upgrading after surgical biopsy of LCIS diagnosed by CNB has been reported by many studies. Nevertheless, the results varied wildly [23–25, 27–29]. In another paper, the authors demonstrated that patients with LCIS had a subsequent 10-year incidence of IBC development of 7.1% [18]. Moreover, the NCCN guidelines 2017 pointed out that surgical removal was suggested once LCIS was diagnosed via CNB [10]. Thus, the large proportion of LA in present study was in line with the current clinical treatment strategy. As for age, analysis demonstrated that it was an irrelevant factor on the choice of treatment options. We also found that MA was most adopted in the white race, which was consistent with a previous study [30]. Our data also revealed that cases with positive ER/PR were 32 times (9.6% vs 0.3%) and 33 times (13.3% vs 0.4%) as many as those with negative ER/PR in NS and LA group respectively. This result indicated that managements with less complication were easier to be accepted among patients with positive ER/PR.

As mentioned above, so far there has been no standardized treatment for LCIS. Some researchers thought that LCIS should be considered as a precursor of ILC, as such, MA was recommended for the treatment [31]. Some others suggested that women diagnosed with LCIS should undergo ipsilateral mastectomy and contralateral breast biopsy based on the results of retrospective studies [32]. Furthermore, there was an increasing trend towards surveillance and chemoprevention alone for LCIS treatment [33, 34]. More recently, a study conducted by Wong et al. demonstrated that women with LCIS often had excellent breast cancer-specific survival and type of surgical treatment for LCIS had no affect on long-term survival [35]. It should be noted that women with LCIS receiving radiotherapy were excluded from analysis in the previous studies.

In our study, both UVA and MVA results indicated that patients underwent LA and LRT had better survival compared to those who accepted other treatments. Although radiation intervention is not recommended for the treatment of LCIS at present [36], the results of our statistical analysis showed that the LRT group indeed had a bit longer average survival time (116.242 months vs. 114.601 months, *P* = 0.317) and smaller HR (0.147 vs. 0.300) than the LA group. Thus, even though no statistical discrepancy was observed between LA group and LRT group, there seems to be a certain clinical benefit for LCIS patients receiving radiation intervention. Further large prospective studies are needed to confirm such benefit of LA and LRT on survival outcomes. Considering the medical expense and the risk of radiotherapy, we think LA may be the most appropriate option for patients with LCIS.

Previous study reported that earlier age at diagnosis and white race were associated with better prognosis [35], which was in accordant with our results. As regards hormone receptor status, positive PR was a beneficial factor for prognosis, while ER status had no significant influence on OS. NCCN guidelines 2017 recommended that chemoprevention should be used for patients with a history of LCIS. NSABP study showed a 56% reduction on the risk of IBC for LCIS patients receiving preventive tamoxifen treatment [34]. Nonetheless, there is still a lack of direct evidence to explain the role of hormone receptors in the prognosis of LCIS. Therefore, more clinical and basic science research is needed to further explore this question.

It was reported that after 12 years’ follow-up, the incidence of subsequent ipsilateral IBC within the population of low and high grade DCIS was 14.4% and 24.6% (*P* = 0.003), respectively [37]. In this study, we found that there was no obvious benefit of histopathological grade on survival, which was very different from DCIS. More research is required to understand the specific biological behavior of LCIS, which in turn may provide useful reference for the clinical management.

There exists some limitations that should be noted in our study. First, information on chemoprevention, which may affect the prognosis of LCIS, is not available in SEER database. Additionally, the possible presence of erroneous data may also serve as an uncertainty on our conclusions. Furthermore, the SEER database is established based on American population, therefore is not able to represent a global situation.

Despite the limitations listed above, to our knowledge, this is the first large population-based study to evaluate impacts of surgical and radiation intervention on survival outcomes of LCIS. It has been very difficult to conduct large prospective studies due to the low incidence of LCIS. The results of our study have certain reference significance on the selection of treatment options of LCIS.

## MATERIALS AND METHODS

### Study cohort and data collection

The National Cancer Institute’s SEER database (http://seer.cancer.gov) contains the publicly available records from 18 cancer registries of 14 states that cover 30% of the US population. SEER.Stat software was used to collect clinical data of women definitely diagnosed with LCIS by pathological examination. The SEER database search was limited to the duration from 2004 to 2013. Cases receiving radiotherapy but not surgical treatment were excluded. Information extracted for each patient included year of diagnosis, age at diagnosis, race, histopathological grade, estrogen receptor (ER), progesterone receptor (PR), treatment, survival months, and vital status. Finally, 16002 cases included were divided into 5 groups according to their treatment as below: (1) No surgery (NS); (2) Lumpectomy Alone (LA); (3) Lumpectomy-Radiotherapy (LRT); (4) Mastectomy Alone (MA); (5) Mastectomy- Radiotherapy (MRT).

### Statistical analysis

Categorical data was analyzed by Pearson’s *Chi* square test. Kaplan–Meier analysis and log-rank testing were used for survival analysis. Cox proportional hazard model was conducted to produce multivariate analysis (MVA) on the basis of univariate analysis (UVA) results. Hazard ratio (HR) was calculated for mortality. All the statistical analyses were performed using SPSS Statistics 22.0. A two-tailed *P* value < 0.05 was used to indicate statistical significance.
